# ASO Author Reflections: The Completion May Lead to Conservation: Partial and Complete Intraoperative Ultrasound Enhanced Breast-Conserving Surgery for Invasive Breast Cancer

**DOI:** 10.1245/s10434-025-18009-x

**Published:** 2025-08-08

**Authors:** Oliver Laszlo Stari, Csaba Torok, Petra Otilia Gorog, Bela Markus, Zoltan Loderer

**Affiliations:** 1 Department of General, Vascular- and Plastic Surgery, Markusovszky Teaching Hospital of Vas County, Szombathely, Hungary; 2 Department of Central Radiology, Markusovszky Teaching Hospital of Vas County, Szombathely, Hungary; 3 Department of Pathology, Markusovszky Teaching Hospital of Vas County, Szombathely, Hungary

## Past

While the widespread adoption of the 2014 SSO-ASTRO Consensus Guideline led to a decrease in reoperation rate, a second surgery is still required in approximately one-tenth to one-fifth of the cases following breast-conserving surgery.^1^ Additionally, a considerable number of experts argue that the definition of a negative surgical margin should be revised.^2^ Altering the “no ink on tumor” standard, however, is likely to affect reoperation rate or lead to unnecessary removal of healthy breast tissue.

Intraoperative ultrasound (IOUS) is an established yet underutilized method for localizing breast lesions. IOUS was also shown to be able to guide tumor resection in real time.^3^ Moreover, it can be used to examine the surgically removed specimen for both tumor involved and close margins.^4^ The versatility of IOUS is undoubtedly a key factor in its effectiveness. On the other hand, this characteristic has also generated substantial differences in IOUS methodology. We retrospectively investigated how the extent of IOUS application affected margin management during lumpectomy for invasive breast cancer.

## Present

In our single-center study the continuous application of IOUS throughout the procedure showed clear superiority over palpation or wire-guided surgery regarding both positive and close resection margins.^5^ While this complete utilization of IOUS led to a significantly more favorable margin status, the extent of breast tissue removal was similar across the study groups. Furthermore, we observed that the rate of tumor involved margins was comparatively low in cases where IOUS was performed solely for tumor localization. However, this partial application of IOUS had minor impact on the rate of close margins.

## Future

A deeper understanding is needed on how the extent of IOUS application affects the outcome of breast-conserving surgery. Future randomized trials on this topic may provide a major contribution to the development and adoption of a standardized IOUS method. Considering that other techniques are available with the capability to enhance margin management, it would also be feasible to compare the effects of IOUS and these methods in future trials.
